# Illustrations of Cutaneous Diseases. A Series of Delineations of the Affections of the Skin, in Their More Interesting and Frequent Forms; with a Practical Summary of Their Symptoms, Diagnosis, and Treatment

**Published:** 1839-07

**Authors:** 


Art. II.—*Illustrations of Cutaneous Diseases. A series of Delineations of the Affections of the Skin, in their more interesting and frequent forms; with a Practical Summary of their Symptoms, Diagnosis, and Treatment.* By Robert Willis, m.d. Fasciculi I. II. III. —*London*, 1839. Folio.

It must be admitted that there is no class of diseases in which pictorial illustration is more useful, or even necessary, than in cutaneous affections. And it cannot be denied that after all the publications of this kind that had taken place, a good work for practical reference, at a moderate price, was still a desideratum when Dr. Willis undertook the superintendence of that before us. We are sorry to say, after examining this work, that the desideratum still exists in a great degree. These illustrations are certainly cheap (four folio plates and as many pages of letter-press being to be had for five shillings), and the plan of the work is strictly practical; but we cannot say that either the plates or the text is good. As works of art, indeed, the plates are very inferior, and the colouring is wretched, generally speaking; while the history and treatment given of the different diseases are meager in the extreme. This latter defect, however, is owing to the faulty plan adopted by the author, not to any incapacity on his part to do the subject justice; and for the execution of the plates we suppose he is not responsible.

With this unfavorable judgment of the work we yet recommend it to our younger readers, as calculated to be of service in enabling them to discriminate the puzzling class of diseases of which it treats. It is cheaper than Bateman's work, and the large size of the figures gives it decided advantages over others of much superior execution, but in which the figures are small, and the affections delineated as it were piecemeal. We had hoped that Dr. Carswell, whose unrivalled work on Pathological Anatomy has carried pictorial illustration to its highest pitch of excellence, would have followed up the publication of that work by another on cutaneous diseases. Had this been done, such a publication as the present would never have been attempted; and should it yet be done, it will soon lose all claim to even the temporary and partial favour which we are disposed to claim for it. We think Dr. Willis's reputation as an author will be somewhat lowered by this work.

